# Centrosome amplification induces high grade features and is prognostic of worse outcomes in breast cancer

**DOI:** 10.1186/s12885-016-2083-x

**Published:** 2016-01-29

**Authors:** Ryan A. Denu, Lauren M. Zasadil, Craig Kanugh, Jennifer Laffin, Beth A. Weaver, Mark E. Burkard

**Affiliations:** Division of Hematology/Oncology, Medical Scientist Training Program and the Department of Medicine, University of Wisconsin School of Medicine and Public Health, Madison, WI USA; Molecular and Cellular Pharmacology Graduate Training Program and the Department of Cell and Regenerative Biology, University of Wisconsin, Madison, WI USA; Wisconsin State Laboratory of Hygiene, University of Wisconsin, Madison, Wisconsin 53706 USA; Department of Cell and Regenerative Biology and University of Wisconsin Carbone Cancer Center University of Wisconsin, Madison, WI USA; Department of Medicine, Division of Hematology/Oncology and University of Wisconsin Carbone Cancer Center, University of Wisconsin-Madison, 6059 WIMR, 1111 Highland Avenue, Madison, WI 53705 USA

**Keywords:** Centriole, Pericentrin, Polyglutamylated tubulin, Chromosomal instability, Polyploidy, PLK4, Dedifferentiated, Mitosis

## Abstract

**Background:**

Centrosome amplification (CA) has been reported in nearly all types of human cancer and is associated with deleterious clinical factors such as higher grade and stage. However, previous reports have not shown how CA affects cellular differentiation and clinical outcomes in breast cancer.

**Methods:**

We analyzed centrosomes by immunofluorescence and compared to ploidy and chromosomal instability (CIN) as assessed by 6-chromosome FISH in a cohort of 362 breast cancers with median clinical follow-up of 8.4 years. Centrosomes were recognized by immunofluorescence using antibodies for pericentriolar material (PCM; pericentrin) and centrioles (polyglutamylated tubulin). CA was experimentally induced in cell culture by overexpression of polo-like kinase 4 (PLK4).

**Results:**

CA is associated with reduced all-cause and breast cancer-specific overall survival and recurrence-free survival. CA correlates strongly with high-risk subtypes (e.g. triple negative) and higher stage and grade, and the prognostic nature of CA can be explained largely by these factors. A strong correlation between CA and high tumor ploidy demonstrates that chromosome and centrosome doubling often occur in concert. CA is proposed to be a method of inducing CIN via aberrant mitotic cell divisions; consonant with this, we observed a strong correlation between CA and CIN in breast cancers. However, some CA tumors had low levels of CIN, indicating that protective mechanisms are at play, such as centrosome clustering during mitosis. Intriguingly, some high-risk tumors have more acentriolar centrosomes, suggesting PCM fragmentation as another mechanism of CA. *In vitro* induction of CA in two non-transformed human cell lines (MCF10A and RPE) demonstrated that CA induces a de-differentiated cellular state and features of high-grade malignancy, supporting the idea that CA intrinsically causes high-grade tumors.

**Conclusions:**

CA is associated with deleterious clinical factors and outcomes in breast cancer. Cell doubling events are the most prevalent causes of CA in cancer, although PCM fragmentation may be a secondary cause. CA promotes high-risk breast cancer in part by inducing high-grade features. These findings highlight the importance of centrosome aberrations in the biology of human breast cancer.

**Electronic supplementary material:**

The online version of this article (doi:10.1186/s12885-016-2083-x) contains supplementary material, which is available to authorized users.

## Background

The centrosome consists of a pair of attached centrioles surrounded by proteinaceous pericentriolar material (PCM) and functions as the major microtubule organizing center in human cells [1]. During interphase, centrosomes organize cytoplasmic microtubules to control cell shape, polarity, and motility; during mitosis, centrosomes separate to form poles of the mitotic spindle. Centrosome aberrations cause human diseases including ciliopathies that arise from mutations in genes encoding centrosome components, such as primary ciliary dyskinesia, autosomal recessive primary microcephaly, polycystic kidney disease, and Bardet-Biedl disease [2]. Furthermore, structural and functional defects of centrosomes are found in cancer, with the most commonly reported being a numerical excess, known as centrosome amplification (CA) [3].

Over a century ago, Theodor Boveri proposed that supernumerary centrosomes can cause cancer [4]. Indeed, CA and other centrosome defects have been reported in diverse cancer types [3, 5]. In breast cancer, centrosome aberrations are common, and amplification correlates with higher tumor grade [6–8], metastasis [9–11], and negative hormone receptor status [12, 13] in small patient cohorts. Yet the causes and consequences of CA in breast cancer remain obscure.

There are several major alternative mechanisms by which CA can arise [3, 5], which we divide into three categories: (1) cell doubling from cytokinesis failure, cell-cell fusion, or endoreduplication resulting in both genome and centrosome doubling; (2) centrosome duplication independent of cell doubling, either *de novo* or due to dysregulation of the centriole cycle; and (3) PCM fragmentation. The relative contributions of these mechanisms of CA to human breast cancer are unclear, but can be addressed with a large cohort of tumor samples. For instance, if polyploidy correlates with CA, this would support genome doubling over centrosome duplication or PCM fragmentation. Moreover, PCM fragmentation is distinguished from duplication in that it is predicted to cause acentriolar centrosomes. Here we evaluate these to provide insight into mechanisms of CA in a large cohort of breast cancers.

The consequences of CA in human cancer also remain unclear. CA is a key mechanism of chromosomal instability (CIN), the perpetual gain or loss of whole chromosomes during cell division. Cells with CA can undergo asymmetric cell division with multipolar spindles, resulting in CIN [6, 14, 15]. CIN leads to large karyotypic diversity among cancer cells, and this genetic diversity provides an enhanced opportunity for selection of highly aggressive clones [16, 17]. Thus, CA can partly explain the karyotypic diversity of breast cancer [18]. However, CA is unlikely to be necessary or sufficient for CIN because CIN can arise from other pathways [19, 20]. Furthermore, cells with CA cluster centrosomes into a pseudo-bipolar spindle under some conditions, allowing them to avoid CIN induced by multipolar division [21]. Prior work has suggested CA is at least partly responsible for CIN in a small cohort of breast cancers [22], but the extent of CA as a cause of CIN is unknown.

In addition to CIN, CA can yield aggressive tumor phenotypes via other mechanisms. For instance, CA causes decreased cilia signaling, altered regulation of Rho GTPases, and increased microtubule-directed polarization [5, 23–25]. Furthermore, CA can behave like an oncogene, increasing cell migration and invasiveness by enhancing Rac1 activity [13, 24]. These ideas suggest that CA may directly promote tumor cell invasion and metastasis without requiring altered genome content. If these preclinical findings operate in human breast cancer, then we would anticipate CA to correlate with altered cancer cell physiology and worse clinical outcomes, independent of CIN.

Here, we assess CA and other centrosome abnormalities and correlate these with FISH data for 6 chromosomes and clinical outcomes in 362 human breast cancers with a median 8.4 years of clinical follow-up. We find that CA portends worse clinical outcomes, and is most prevalent in high-risk breast cancer. The data suggest that multiple mechanisms contribute to the development of supernumerary centrosomes and that CA promotes aneuploidy. There is a strong correlation between CA and tumor grade, providing a potential mechanism for the aggressive behavior of high-grade tumors. Accordingly, in cell models, induced CA promotes expression of cellular markers of de-differentiation and induces high-grade phenotypes. These findings provide important insight into how CA arises and how it imparts high-grade phenotypes and worse clinical outcomes in human breast cancer. Moreover, our findings suggest that pharmacologic interventions on CA or its downstream effects could improve outcomes for patients with centrosome-amplified cancers.

## Methods

### Patients, tissues, ethics, and consent

The breast cancer tissue microarray (TMA) used in this analysis has been described previously [26, 27]. Briefly, samples were obtained from primary breast tumor blocks obtained at time of surgery for stage I-III breast cancer patients seen at the University of Wisconsin Carbone Cancer Center under protocol OS10111. The University of Wisconsin Health Sciences Institutional Review Board approved the TMA creation and approved use of the TMA and the de-identified coded data set (IRB approval 2010-0405). This protocol retrospectively collected de-identified data and archived tissue; the IRB waived patient consent. The TMA contains three 0.6 mm punch biopsies from each patient’s tumor, and 15 normal breast controls from mammoplasty are included in the array. All cases had at least 5 years of follow-up or recurrence or death within 5 years. Clinical information includes age at diagnosis, ethnicity, tumor size, lymph node involvement, stage, estrogen receptor (ER), progesterone receptor (PR), and HER2 status, type of surgery, adjuvant breast cancer treatments, and follow-up data, including any recurrence and death. Clinical data was obtained from the UW Hospital and Clinics Cancer Registry and manual chart review. ER, PR, and HER2 immunohistochemistry were also performed on the completed TMAs and interpreted by a breast pathologist. If ER/HER2 clinical data was not available, the clinical pathologic data from the original tumor sample was used for analysis. Patients with unknown or equivocal values were excluded from these analyses of subtype and CA. For subtype analysis, the following groups were used based on their clinical relevance [28, 29]: ER or PR positive and HER2-nonamplified; HER2-amplified; and triple negative.

### Immunohistochemistry

Breast cancer TMAs were sectioned at 5 μm thickness, deparaffinized, and rehydrated. Antigen retrieval was performed in a pressure cooker at 250 **°**F with citrate buffer (pH 6) for 4 minutes. Blocking was done for 1 hour in 10 % fetal bovine serum (FBS) in PBS. Tissues were probed with anti-pericentrin (Abcam, ab4448, 1:200) and anti-polyglutamylated tubulin (Adipogen, GT335, 1:100) antibodies diluted in 1 % FBS and 0.1 % triton X in PBS overnight in a humidified chamber at 4 **°**C. Pericentrin and polyglutamylated tubulin are *bona fide* markers of centrosomes [30–32]. The TMAs were then incubated with anti-rabbit Alexa 488 and anti-mouse IgG1 Alexa 647 secondary antibodies (Jackson ImmunoResearch Laboratories, West Grove, PA) for 1 hour at room temperature. Slides were washed 3 times after primary and secondary antibody incubations. Slides were counterstained for DNA with 4′,6-diamidino-2-phenylindole (DAPI) and mounted with ProLong Gold antifade reagent (Life Technologies). Scoring of centrosome phenotypes was performed using a Nikon Eclipse Ti inverted microscope, 100x objective, and CoolSNAP HQ2 charge-coupled device camera (Photometrics). The observer was blinded to clinical data and analyzed centrosomes in a minimum of 30 cells per case from 3 different tumor regions. The number of distinct pericentrin foci as well as foci that overlapped with polyglutamylated tubulin were counted. Cell boundaries were visualized by nonspecific background staining with the polyglutamylated tubulin antibody. Average centrosome number per cell was calculated for each case. Centrosome sizes were measured in at least 15 representative centrosomes per case from three different tumor regions using the pericentrin marker, and an average was calculated for each case. For survival analysis, the median centrosome size (0.99 μm) was used as the cutoff for large versus small centrosomes. In addition to number and size, we also noted any unusual centrosome phenotypes such as centrosome clustering, centrosome speckling, and atypical shapes.

A small fraction of samples in the TMA were not evaluable due to loss of tissue, insufficient cellularity, or other technical issues and were excluded from analysis. Centrosome data were linked to de-identified clinical data by sample number and position on the TMA and sorted for analysis using Microsoft Excel.

### Fluorescence in situ hybridization

Fluorescence in situ hybridization (FISH) was performed using standard techniques, as reported elsewhere [33]. Briefly, chromosomes 4, 10, and 17 were probed on one section, and chromosomes 3, 7, and 9 on another section. Chromosomes were counted by observers blinded to patient conditions in a minimum of 10 cells per case. A small fraction of samples were not evaluable due to loss of tissue, insufficient cellularity, or other technical issues and were excluded from analysis. Similarly a subset of samples had a single probe that was not well visualized, but if at least five chromosomes were available, it was included in further analyses. FISH data were linked to de-identified clinical data by sample number and position on the TMA and sorted for analysis using Microsoft Excel. Ploidy was determined by the average chromosome number for all 6 probes combined. CIN was determined as the average percentage of cells that deviated from the modal number for each of the 6 chromosomes assessed by FISH. Samples were considered to have CIN if this value exceeded 45 %, a cutoff that yielded appropriate percentages of normal samples and tumors with CIN.

### Cell culture

The doxycycline-inducible PLK4^WT^ and PLK4^608^ MCF10A and RPE cell lines were a kind gift from Dr. David Pellman. Cells were cultured and centrosome amplification was induced as previously described [24, 33]. For assays, cells were treated with 2 μg/mL doxycycline for 48 hours and subsequently harvested for qRT-PCR and flow cytometry. Immunofluorescence was performed as previously described using the following antibodies: anti-pericentrin (Abcam, ab4448), anti-gamma tubulin (Abcam, ab27074), anti-alpha tubulin (Millipore, MAB1864), and Alexa fluorophore-conjugated secondary antibodies (Jackson).

### Quantitative reverse transcriptase polymerase chain reaction (qRT-PCR)

RNA was isolated from cells using the RNeasy Micro Kit (Qiagen, Valencia, CA), and converted to cDNA using the Quantitect Reverse Transcription Kit (Qiagen). qRT-PCR was performed using EvaGreen master mix (MidSci, St. Louis, MO) and a StepOne Plus instrument (Applied Biosystems). Quantification of cytokeratins 7, 18, and 19 (*KRT7, KRT8, KRT19*) expressed as mRNA level was normalized to the mRNA of three housekeeping genes (*RRN18S, GAPDH and ACTB*). Primers sequences are provided in Additional file 1: Table S3. Fold changes in gene expression were assessed using the 2^-ΔΔCt method [34].

### Flow cytometry

A total of 50,000 events were acquired for each sample using an Accuri C6 flow cytometer (Accuri, Ann Arbor, MI) equipped with multicolor analysis, and data were analyzed with Flow Jo 7.0 (Tree Star, Ashland, OR). Samples were run in triplicate in at least 3 independent experiments. The following antibodies were used: CD24-PE, CD44-PE, and mouse IgG1 isotype control (BD Biosciences). Mean channel fluorescence of FL2 was used to quantitatively compare conditions.

### Statistical analysis

R (version 3.1.1, R Core Team, Vienna, Austria) statistical software was used for survival analysis. A total of 362 patients were included in survival analyses. The clinical outcomes analyzed in this study were recurrence-free survival (RFS) and overall survival (OS). RFS was defined as the time from initial breast cancer diagnosis to recurrence. OS was defined as the time from diagnosis to the date of death. RFS and OS were plotted using the Kaplan–Meier method, and log-rank tests were used to compare patients with tumors with CA versus tumors with no CA using 2 centrosomes per cell as a cutoff. Sensitivity analyses were also performed using the mean and median of all the normal breast samples in the TMA as the cutoff for defining CA. Cox proportional hazards model included centrosome amplification, stage, tumor grade, hormone receptor status, and HER2 status. Associations between these factors and either RFS or OS were analyzed and presented as hazard ratios (HR) with 95 % confidence intervals (CI). For centrosome size, an average size was calculated for each case. The median of all cases was used as the cutoff for the large versus small centrosome groups. The correlations of CA with ploidy and CIN were assessed with Spearman’s correlation. The correlations between centrosome amplification and grade or stage were performed by stratifying patients by grade or stage and comparing the mean centrosome number among the groups by Kruskal-Wallis tests. Non-parametric tests were used because the distribution of average centrosome number was right skewed (Additional file 1: Figure S1). Two-sided, unpaired statistical tests were used throughout. *P* < 0.05 was considered statistically significant for all statistical tests.

## Results

### Centrosome amplification is associated with adverse clinical factors and worse survival

**Fig. 1:**
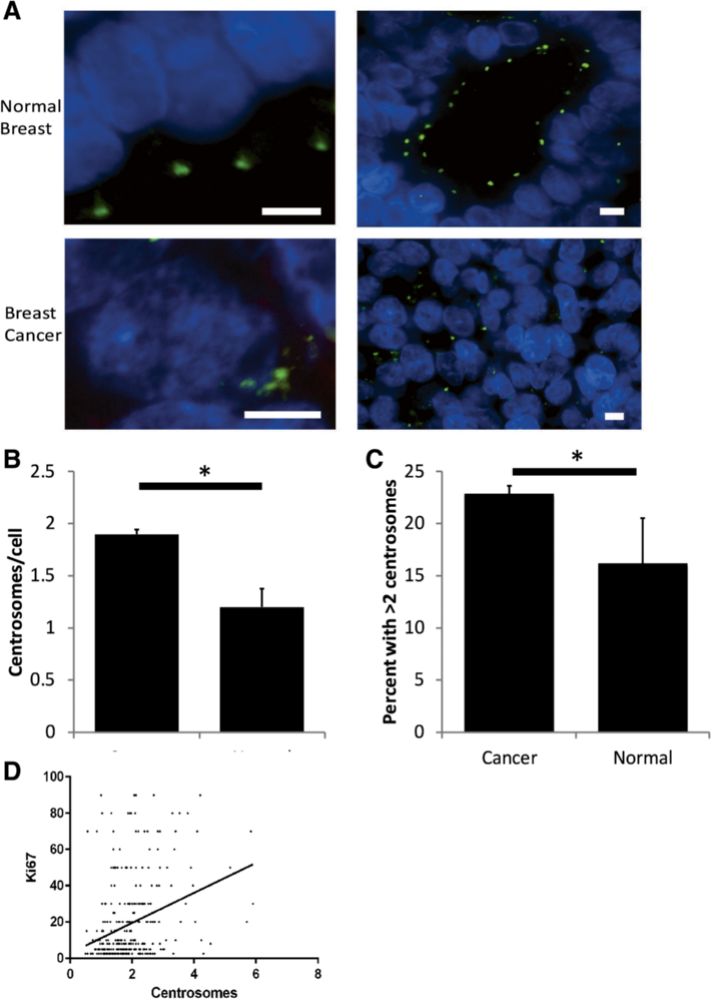
Centrosome amplification in breast cancer. **a** Representative single plane images of normal breast and breast cancer from the TMA taken using the 100x objective. Blue = DNA, green = pericentrin. Scale bar = 5 μm. **b** The average centrosome number per cell (as assessed by pericentrin staining) is significantly greater in breast tumors than in normal breast samples included in the TMA (*p* = 0.0012 from unpaired, two-tailed t-test). **c** The percentage of tumors with an average of > 2 centrosomes per cell is significantly greater than the percentage of normal breast samples with > 2 centrosomes per cell in the TMA (*p* = 0.049). **d** Scatterplot demonstrating the correlation between average centrosome number and proliferation, as assessed by Ki67 staining

We initially characterized the distribution of centrosome abnormalities and clinical characteristics seen in our breast cancer samples. Patient characteristics are shown in Additional file 1: Table S1. Centrosomes were assessed in each sample using pericentrin, a PCM marker. The normal mammary gland is composed of terminal ductal lobular units, and polarity is well defined, as indicated by luminal positioning of the centrosome and basal positioning of the nucleus (Fig. 1a, top). However, this organization is disrupted in carcinoma samples (Fig. 1a, bottom). Furthermore, the median centrosome number from all the tumor samples was almost double that of the normal breast samples (1.8 vs. 1.0, *p* = 0.001), and 84 % (305 of 362) of breast cancer samples had a mean centrosome value higher than that of the normal breast samples. Additionally, the average centrosome number per cell and the percent of cells with greater than 2 centrosomes were both significantly greater in breast cancers compared to normal breast (Fig. 1b-c). To demonstrate that this is not simply due to a greater proliferative rate in the tumors (as centrosomes are duplicated at the G1/S transition), we correlated average centrosome number with Ki67, a marker of proliferative index. Although there is a partial correlation, a significant portion of samples have high centrosome number with low Ki67 (Fig. 1d). Because of the partial correlation, an elevated average centrosome number between 1 and 2 could indicate an increase in the percent of tumor cells in G2. Hence, we used a strict cutoff of >2 centrosomes for subsequent analyses of CA. Breast cancers also showed a wider distribution of mean centrosome number per cell compared to normal breast (range 0.5–5.9 and 0.23–2.77, respectively), consistent with CA occurring in a variable fraction of cells within a tumor. The distribution of centrosomes in breast cancer was unimodal with right skew, while the distribution of centrosomes in normal breast samples was normal (Additional file 1: Figure S1).

**Fig. 2:**
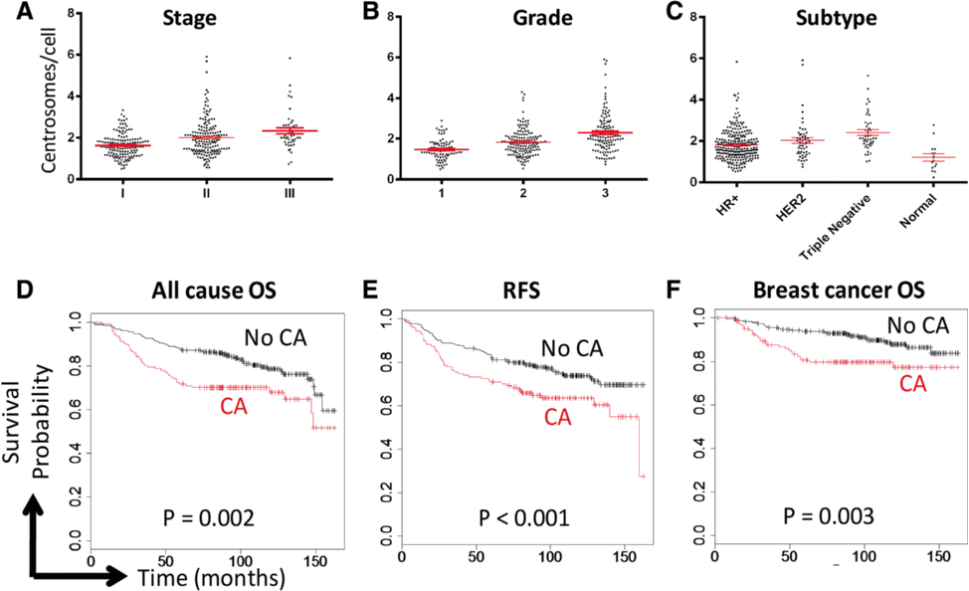
Centrosome amplification is associated with adverse clinical factors. Centrosome amplification correlates with stage (**a**; *p* < 0.01), grade (**b**; *p* < 0.01), and subtype (**c**; *p* < 0.01). Dots represent each patient with bars representing the average ± SE. HR = hormone receptor. **d**-**f** Tumors were considered to have CA if the average number of centrosomes per cell, as assessed by pericentrin staining, was greater than 2. Centrosomes were assessed with pericentrin staining. All-cause overall survival (**d**), recurrence-free survival (**e**), and breast cancer-specific overall survival (**f**) are reduced in patients whose tumors demonstrated CA compared to those that did not. Log rank tests were used to calculate *p*-values

We stratified patients based on stage, grade, subtype, regional node status, and recurrence site. Patients with higher stage and grade also had a higher average number of centrosomes per cell (Fig. 2a-b). Furthermore, CA was greater in triple negative and HER2 amplified subtypes (Fig. 2c); in general, estrogen/progesterone receptor-positive breast cancers have a more favorable prognosis than HER2 amplified or triple negative cancers [35].

A tumor was considered to have CA if the mean number of pericentrin foci per cell exceeded 2. Using this definition, CA was found in 35.1 % of breast tumors and 13.3 % of normal breast samples. It was most common in triple negative breast cancers (61.4 %) and less frequent in HER2-positive (41.2 %) and hormone-sensitive/HER2-negative subgroups (29.2 %). We next assessed how CA correlated with clinical outcomes. Patients with CA had significantly worse overall survival (OS, *P* = 0.002; Fig. 2d) and recurrence-free survival (RFS, *P* < 0.001; Fig. 2e) than those without CA. Furthermore, these patients also had worse breast cancer-specific mortality (*P* = 0.003; Fig. 2f). Our findings led us to hypothesize that high-CA tumors may provide useful prognostic data in addition to providing a biologic reason for aggressive breast cancers. To be clinically useful, CA would need to indicate risk that is not captured with currently available clinical factors such as tumor stage, grade, and subtype. To test this, we performed Cox proportional hazards modeling (Additional file 1: Table S2). This analysis demonstrated that stage and hormone receptor status were the strongest predictors of OS and RFS. When corrected for these, CA is not an independent predictor of OS or RFS. Although CA does not provide a clinical factor independent of known risk factors, it nevertheless may provide a biological explanation for how tumors advance in grade and stage.

To survey additional centrosome defects, we observed aberrations in centrosome shape, size, and patterning (Additional file 1: Figure S2). Centrosome clustering was observed in 58 % of tumors versus 13 % of normal samples. Centrosome speckling (clusters with >5 centrosomes) was observed in 23 % of tumors versus 7 % of normal samples, and irregular centrosome shapes in 41 % of tumors versus 20 % of normal samples (this relatively high incidence of abnormal shapes in normal samples likely represents staining artifact). What we term centrosome speckling has been described by others as sand-like centrosomes [12]. We did not observe worse clinical outcomes with atypically shaped centrosomes or centrosome speckling, although centrosome clustering correlated with significantly worse OS (*P* = 0.009) and RFS (*P* = 0.030). Centrosome clustering has been proposed as a mechanism by which cells with CA are able to divide with pseudo-bipolar spindles [36, 37], although it is unclear whether the interphase clustering observed here would correspond with clustering during mitosis.

### Doubling events as a common cause of centrosome amplification

**Fig. 3:**
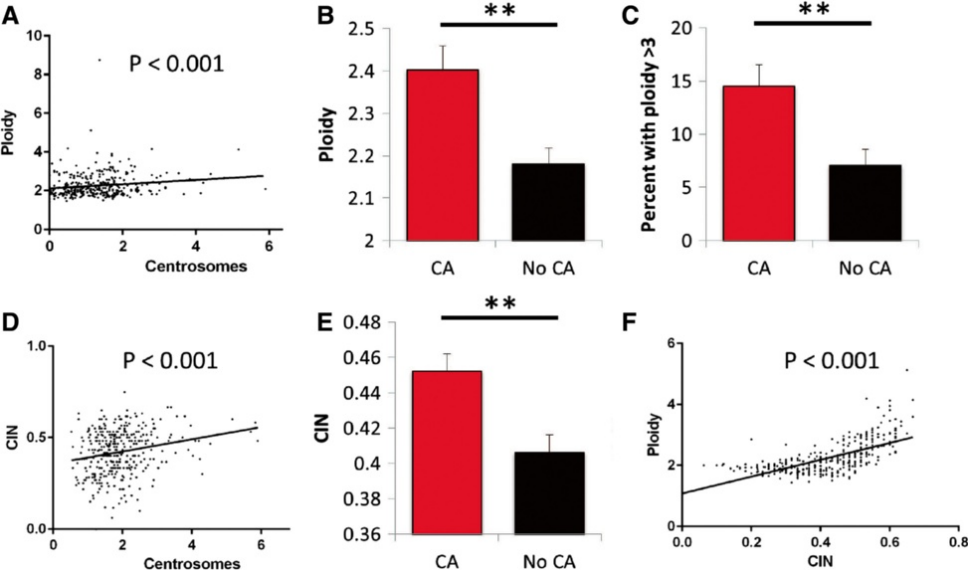
Centrosome amplification correlates with high ploidy and high CIN. **a** Centrosome number correlates with ploidy. **b**-**e** Breast tumors were divided into two groups based on the presence or absence of CA, which was defined as having an average of >2 centrosomes per cell, as in Figure 2. Tumors with CA had higher average ploidy, as assessed by 6-chromosome FISH (**b**), polyploidy (**c**), and CIN, as assessed by the average non-modal chromosome number from FISH (**e**). **d** Centrosome number correlated with CIN. **f** Ploidy and CIN also correlated.

One potential mechanism leading to CA is cell-doubling events (e.g. cytokinesis failure, cell-cell fusion). If cell doubling represented the primary cause of CA in breast cancer, we would expect a strong correlation between CA and increased cell ploidy. Therefore, we evaluated how CA correlates with high tumor ploidy, as determined by 6-chromosome FISH in 354 breast tumors. Ploidy ranged from 1.43 to 8.75 with a median of 2.08. We find that CA strongly correlates with ploidy (*P* = 0.006; Fig. 3a). Further, after dividing patients by CA (defined as >2 centrosomes per cell), tumors with CA had significantly greater ploidy (Fig. 3b). To verify these findings, analyses were repeated using a more stringent definition for centrosomes: the overlap of pericentrin and polyglutamylated tubulin, which represents the overlap of PCM and centriole markers, respectively [30, 31]. Using these criteria, CA still correlated with ploidy (Additional file 1: Figure S3). These data provide evidence that CA and whole genomic amplifications occur in concert in incipient tumor cells, suggesting that genome doubling events occur commonly in breast cancer oncogenesis.

To estimate what percentage of CA events arise from doubling events, we calculated the percent of tumors with CA (average centrosome number >2) that also had elevated ploidy (>3). This revealed that at least 15 % of CA events arose from doubling events (Fig. 3c). However, this method is likely to underestimate the true percentage of CA events that arise from doubling events because cells that originate after genome doubling can subsequently lose chromosomes [38, 39].

### Centrosome amplification as a common cause of chromosomal instability

CA can lead to multipolar cell division or lagging chromosomes through induction of merotelic attachments on focused bipolar spindles, resulting in chromosomal instability (CIN) [39]. Therefore, we examined the relationship between CA and CIN. CIN was calculated as the percent of cells within a tumor with a non-modal number of chromosomes, averaged for 6 chromosomes. 44.7 % of breast tumors have CIN compared to 9.1 % of normal breast samples. Patients whose tumors displayed CIN had worse breast cancer-related overall survival (Additional file 1: Figure S3). CA correlated positively with CIN (Fig. 3d, *P* < 0.001). After dividing the patients into two groups based the presence of CA, as done for survival analyses, tumors with higher CA had significantly elevated CIN (Fig. 3e). These data support the hypothesis that CA is a common cause of CIN in breast cancer. In addition, we found a strong positive correlation between ploidy and CIN (Fig. 3f).

### Pericentriolar material fragmentation is a marker of aggressive tumors

As done above for CA and ploidy analysis, we repeated other analyses using the more stringent definition of pericentrin and polyglutamylated tubulin overlap. Similar to our analysis based on pericentrin staining alone, CA defined by the overlap of pericentrin and polyglutamylated tubulin was more pronounced in triple negative breast cancer and cancers with higher histological grade (Additional file 1: Figure S4A-D). Patients with CA had worse overall and recurrence-free survival (Additional file 1: Figure S4E-G). Furthermore, CA as defined by these criteria also demonstrated a significant correlation with ploidy and CIN (Additional file 1: Figure S5A-D). In summary, we observed similar findings whether centrosomes were defined using solely pericentrin or using the overlap of pericentrin and polyglutamylated tubulin.

**Fig. 4:**
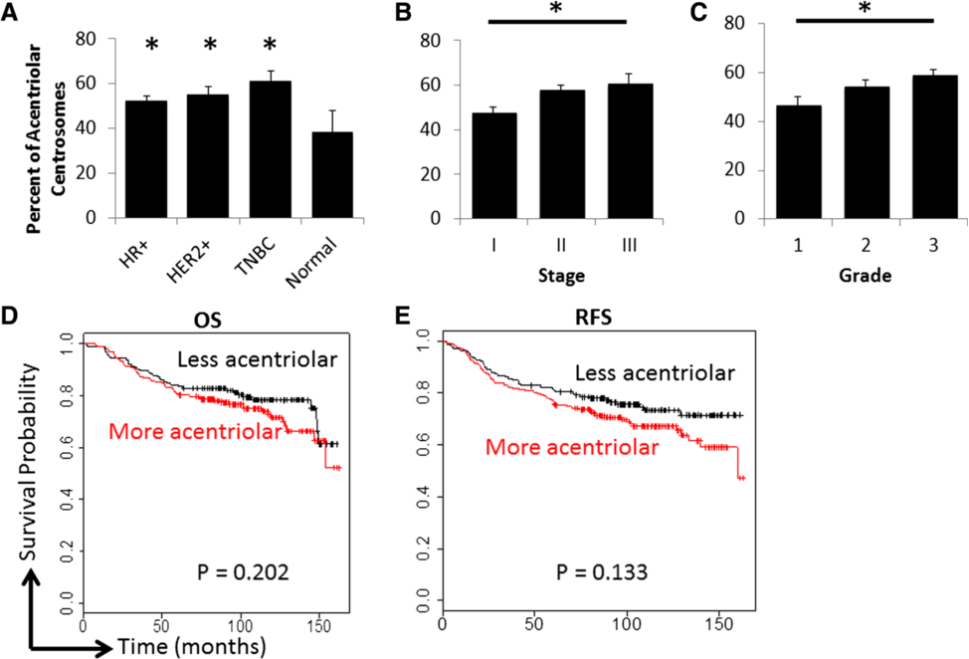
Pericentriolar material (PCM) fragmentation is more prevalent in advanced tumors. **a**-**c** The average percent of centrosomes (as indicated by pericentrin) without centrioles (as indicated by polyglutamylated tubulin) were plotted based on subtype (**a**), stage (**b**) and grade (**c**). **a** T-tests were used to compare averages for each breast cancer subtype to normal breast. HR+ = hormone receptor (ER and/or PR) positive and HER2 nonamplified; HER2+ = HER2 amplified; TNBC = triple negative breast cancer. **b**-**c** ANOVA was used to analyze differences across stage and grade; the asterisk indicates *P* < 0.05 for these statistical tests. **d**-**e** Overall survival (**d**) and recurrence-free survival (**e**) were plotted using the Kaplan Meier method with the cutoff being the median percentage of centrosomes without centrioles. Log rank tests were used to determine P values

Although centrioles are surrounded by PCM in normal cells, ~1/3 of cells in normal samples had PCM without detectable centrioles, suggesting that only a subset of centrioles were labeled with the polyglutamylated tubulin antibody. However, compared with normal samples, tumors more frequently had pericentrin foci that lacked co-staining with polyglutamylated tubulin. An average of 78 % of pericentrin foci contained this centriolar marker in normal samples compared to an average of 46 % in breast tumors. 302 out of 362 breast cancer cases had a percentage lower than 78 %, suggesting a true loss of this centriole marker in some breast tumors. These findings suggest that either these tumor centrioles lack polyglutamylated tubulin, or that acentriolar centrosomes are a *bona fide* characteristic of many human breast cancers. Acentriolar centrosomes have been reported previously in cancer cells and are thought to result from PCM fragmentation [19, 40]. Additionally, acentriolar centrosomes were more common in the triple-negative breast cancer subtype and correlated with advanced stage and grade (Fig. 4a-c), although there was no significant correlation with worse clinical outcomes (Fig. 4d-e; *P* = 0.202 for overall survival and *P* = 0.133 for recurrence-free survival). Nevertheless, these findings indicate that PCM fragmentation is potentially a marker of more aggressive tumors.

### Centrosome amplification causes high-grade features

**Fig. 5:**
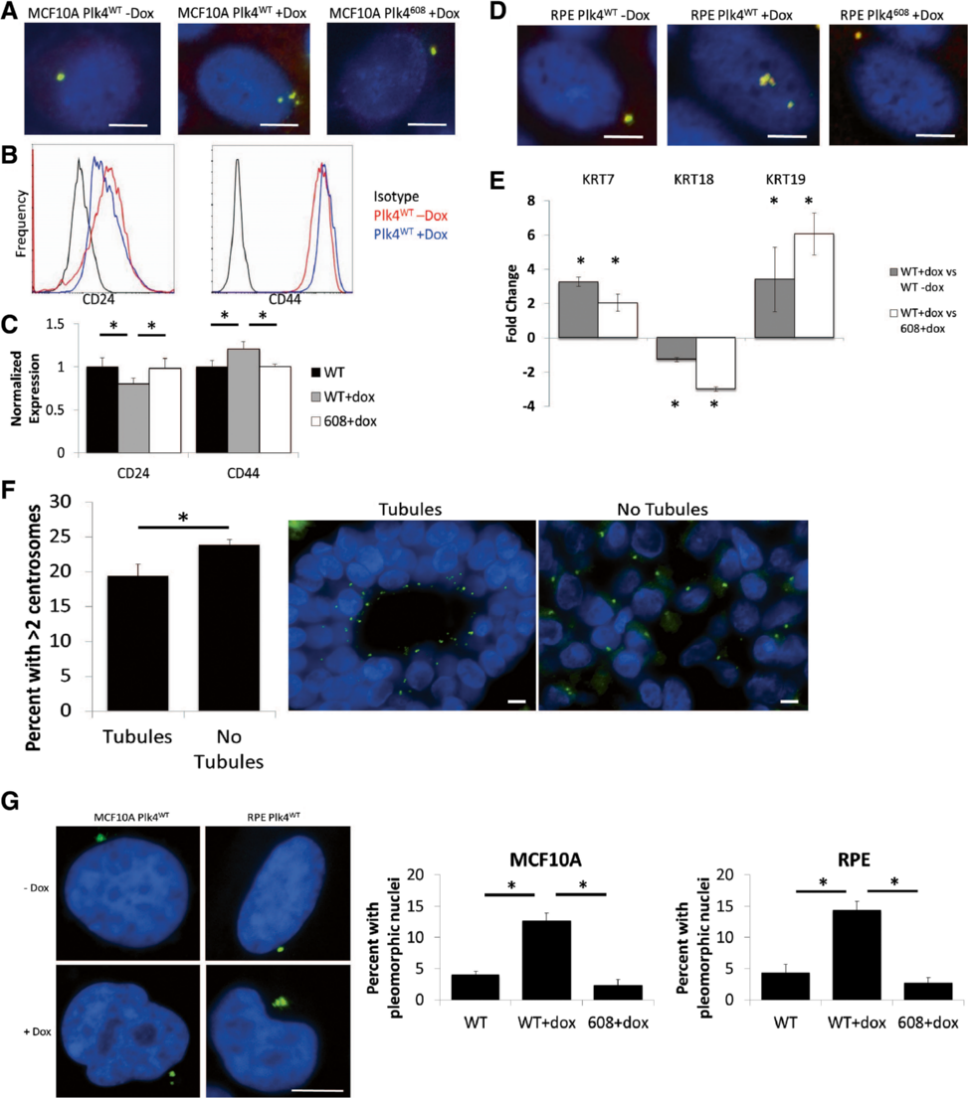
Centrosome amplification induces dedifferentiation. **a** Overexpression of PLK4 using doxycycline-inducible MCF10A cell line [24] results in CA after 48 hours of doxycycline treatment. PLK4^608^ overexpresses a truncated form (amino acids 1-608) that contains the kinase domain but does not result in CA. Blue = DNA, yellow = overlap of pericentrin and gamma tubulin. **b**, **c** Flow cytometry analysis of CD24 and CD44 expression in MCF10A cells using PE-conjugated antibodies after 48 hours of doxycycline treatment. **b** Mean channel fluorescence (FL2) was normalized within cell lines (i.e. either PLK4^WT^ or PLK4^608^). **c** Bars represent the average ± SE of 3 independent experiments. **d** Overexpression of PLK4 using doxycycline-inducible RPE cell line [41] results in CA. **e** qRT-PCR analysis of cytokeratins 7, 18, and 19 normalized to 3 housekeeping genes (*RRN18S, GAPDH, ACTB*) in RPE cells. Bars represent average values of 2^-∆ΔCt from 3 independent experiments. **f** Tumors in the TMA with tubule formation in 2 of the 3 tumor regions examined were compared to tumors without tubule formation. Representative images demonstrate how the scoring was performed. Blue = DNA, green = pericentrin. **g** Representative images of normal and pleomorphic nuclei seen in RPE and MCF10A cell lines treated with doxycycline. The bar graphs demonstrate the average percent of pleomorphic nuclei in each condition from 3 independent experiments. Blue = DNA, green = pericentrin. *P < 0.05. Scale bars = 5 μm

Because there was a strong correlation of CA with poorly differentiated tumors (grade 3) in our study and others [6–8], we hypothesized that CA induces cellular de-differentiation. To test this, we utilized doxycycline-inducible PLK4 in MCF10A and RPE cell lines [24, 41], in which the overexpression of PLK4 results in CA (Fig. 5a, d), and subsequently looked at markers of differentiation. Breast cancer cells that express less CD24 and more CD44 are more de-differentiated and more stem cell-like [42–44]. These cells may also have enhanced metastatic potential [45]. We analyzed CD24 and CD44 by flow cytometry after inducing CA in MCF10A cells, and found that this significantly decreased CD24 and increased CD44 (Fig. 5b-c). To ensure this was not an effect of doxycycline or of PLK4 expression independent of centrosome amplification, we employed a doxycycline-inducible PLK4^1-608^ cell line in which this kinase is expressed without amplifying centrosomes due to lack of a critical localization domain. We did not observe markers of de-differentiation with doxycycline in this control (Fig. 5c). To validate this finding, we employed a second cell line, immortalized retinal pigment epithelial (RPE) cells, for which cytokeratin profiles can reveal differentiation status. More de-differentiated cells express excess cytokeratins 7 and 19 and less cytokeratin 18 [46–49]. We assessed expression of these 3 cytokeratins by qRT-PCR, finding that RPE cells with CA express more cytokeratins 7 and 19, but less cytokeratin 18 (Fig. 5e), which is consistent with a de-differentiated state.

The Nottingham grading scale uses the following three criteria to determine the differentiation status of a tumor: (1) the amount of gland formation, (2) nuclear pleomorphisms, and (3) mitotic figures [50, 51]. To address whether CA is sufficient to impart these characteristics, we first observed which tumors demonstrated glandular/tubular structures in at least two of three histologic regions examined. Indeed, tumors from the TMA without tubule formation had greater CA (Fig. 5f). With regard to the second criterion, previous work has shown cells with CA can exhibit multipolar spindles and other nuclear pleomorphisms, and this is seen *in vitro* as well (Fig. 5g). For the third criterion, it has already been demonstrated that cells with CA proliferate more slowly [24, 52], which would tend to cause lower grade tumors; however, numerical CA positively correlates with Ki67 status in our data set (Pearson *r* = 0.3106, *p* < 0.001, Fig. 1d) suggesting that CA does not cause tumor cells to exit the cell cycle. Furthermore, it has been demonstrated that cells with CA take longer to complete mitosis due to multipolar spindle formation [53], which could explain why more mitotic figures are seen in tumors with CA. Taken together, these data support the idea that CA directly or indirectly imparts high-grade features to tumors, leading to worse clinical outcomes.

## Discussion

Our findings provide important insight into the origin, frequency, and the clinical correlates of CA in human breast cancer. CA was previously reported in small sample sizes to be a hallmark found in diverse cancer types [3], and is often found early in carcinogenesis, including in precursor lesions of breast cancer [22]. Likewise, we find that CA is common in our cohort of 362 breast cancer patients. CA correlates with increasing grade and stage, and CA was more pronounced in triple negative and HER2 amplified subtypes, consistent with past observations in smaller patient cohorts [6–13]. Further, CA confers worse outcomes, which can be explained by the aggressive characteristics of cancers with CA, including advanced stage and grade. Intriguingly, CA is sufficient to induce high-grade phenotypes in human epithelial cells, including those of breast origin. This can explain why tumors that originate with CA have de-differentiated phenotypes that presage worse clinical outcomes. Additionally, CA can cause CIN, leading to rapid evolution of tumors into more aggressive phenotypes.

Our data provide the first evidence for the origin of CA in breast cancer. The data indicate that at least 15 % of cases of CA in human breast cancer arose by a doubling event, such as cytokinesis failure or cell-cell fusion. However, our method likely underestimated the true percentage of CA from doubling events because CA can lead to further chromosome loss, and tetraploidy buffers the risk of haploinsufficiency [38, 39]; hence some CA cancers with near-diploid genomes could have originated in a doubling event. To more definitively answer this question regarding the relative contributions of mechanisms leading to CA, a large tumor cohort could be probed for a marker of mature centrioles, such as CEP170 or centrobin. Cells with *de novo* centrosome amplification should have a single CEP170-positive centrosome, whereas tumor cells with multiple CEP170-positive centrosomes are more consistent with doubling events, in which two mature centrosomes would be inherited.

PCM fragmentation has been proposed as a mechanism by which CA can occur [19, 40, 54]. Indeed, we found centrioles absent from a sizeable proportion of centrosomes in the tumor samples in our TMA. This suggests that the regulation of recruitment of centrosome proteins is just as important as the regulation of centriole duplication for proper centrosome function. Excess PCM or PCM fragmentation may result in cells that are likely to undergo multipolar mitoses and generate daughter cells with altered karyotypes. Many of these daughter cells generated from multipolar divisions will not be viable but will promote diversity for evolutionary selection. We found that an increased percentage of acentriolar centrosomes correlated with adverse clinical features, suggesting that PCM fragmentation is more common in more aggressive tumors.

Previous studies have provided conflicting information about how centrosome number and size correlate with aneuploidy and CIN in breast cancer, with some studies supporting the relationship [22], and others finding no association [55]. Here we quantified a greater number of chromosomes with FISH on a larger patient cohort and found a strong correlation of CA with both CIN and aneuploidy. This is consistent with mechanistic studies that illustrate that CA can cause CIN and aneuploidy [6, 14, 15]. The strong correlation between CA and ploidy suggests that CA can occur in many breast cancers from prior cell doubling events, although it is unclear whether this originates from failed cell division, cell-cell fusion, or other mechanism such as endoreduplication. Furthermore, we found a strong positive correlation between ploidy and CIN, consistent with the previous suggestion that cells with higher ploidy can better tolerate CIN and buffer the deleterious effects of CIN [38]. However, there were a number of cases (approximately 18 %) in which CIN did not correlate with CA. First amongst these was a high CA, low CIN group, in which centrosome clustering may be occurring; clustering has been described previously as a way that the cell prevents multipolar spindle formation [21, 36, 56], although this can still result in chromosome missegregation [39]. In a second group with low CA and high CIN, another mechanism of generating CIN is operating, such as impaired checkpoint function [19–21].

Advantages of our methods include a large cohort of breast tumor samples with survival data, evaluation of 6 chromosomes by FISH for analysis of aneuploidy and CIN, and use of the overlap of PCM and centriole markers to characterize acentriolar centrosomes and PCM fragmentation. This allowed us to establish how CIN and ploidy correspond with centrosome number in hundreds of breast tumors. A potential limitation to this study is dependence on single-section analysis of histological samples, which underestimate CA and overestimate CIN through sectioning artifact. However, the inclusion of triplicate punch biopsies per patient and normal breast samples help to alleviate this concern. It is possible that larger cancer cells could suffer disproportionate underestimation of CA than normal cells; however this does not explain our finding that CA is commonly found in larger high-grade tumor cells. Nevertheless, our results provide quantitative comparisons among breast cancer types and shed important insight into the causes and consequences of CA in human breast cancer.

## Conclusions

CA is a common feature of human breast cancers that presages worse clinical outcomes but is not an independent predictor of survival. CA arises by multiple mechanisms, most predominantly by doubling events and PCM fragmentation. PCM fragmentation may represent a marker of high-risk cancers. In human cancer CA is associated with a high-grade phenotype and loss of genetic stability. These factors lead to the aggressive phenotypes of cancers with high CA. It may be possible to interrupt these phenotypes with specific drugs targeting centrosome amplification, such as recently discovered inhibitors of PLK4 [57–59].

### Availability of supporting data and materials

All of the data on which the conclusions of the paper rely are included in the main figures or supplementary figures.

## Additional file

Additional file 1:
**Table S1.** Patient characteristics. **Table S2.** Hazard ratios from multivariate analysis. **Table S3.** Sequences of primers used for qRT-PCR. **Figure S1.** Distribution of average centrosome number per cell in the breast cancer patients represented in our TMA. **Figure S2.** Correlations between centrosome amplification and nodal status, patient age, and tumor size. **Figure S3.** Centrosome clustering but not structural abnormalities correlate with worse outcomes in breast cancer. **Figure S4.** CIN is prognostic of worse breast cancer-related survival. **Figure S5.** Centrosome amplification correlates with adverse clinical factors. **Figure S6.** CA correlates with higher ploidy and CIN. (DOCX 6223 kb)

## Abbreviations

CA: centrosome amplification
CIN: chromosomal instability
HER2: human epidermal growth factor receptor 2
HR: hormone receptor
OS: overall survival
PCM: pericentriolar material
RFS: recurrence-free survival
TNBC: triple negative breast cancer
