# Supplementary material for: Centrosome amplification induces high grade features and is prognostic of worse outcomes in breast cancer
Source: BMC Cancer. 2016 Jan 29;16:47. doi: 10.1186/s12885-016-2083-x (PMC4734858; doi:10.1186/s12885-016-2083-x)

**Supplementary Material**

**Centrosome Amplification Induces High Grade Features and is Prognostic of Worse Outcomes in Breast Cancer**

Supplemental Table S1: Patient characteristics

| **Characteristic** | **Number** | **Percent** |
| --- | --- | --- |
| Race |  |  |
| Caucasian | 348 | 96.1% |
| Black | 6 | 1.7% |
| Asian | 3 | 0.8% |
| Hispanic | 2 | 0.6% |
| Unknown/Other | 3 | 0.8% |
| Sex |  |  |
| Female | 362 | 100.0% |
| Male | 0 | 0.0% |
| Age at diagnosis |  |  |
| <40 | 37 | 10.2% |
| 40-49 | 101 | 27.9% |
| 50-59 | 96 | 26.5% |
| 60-69 | 64 | 17.7% |
| 70-79 | 44 | 12.2% |
| >=80 | 20 | 5.5% |
| Histology |  |  |
| Ductal or ductal subtype | 301 | 83.1% |
| Lobular | 33 | 9.1% |
| Mammary | 26 | 7.2% |
| Phyllodes | 1 | 0.3% |
| Adenoid cystic | 1 | 0.3% |
| Histological Grade |  |  |
| 1 | 83 | 22.9% |
| 2 | 149 | 41.2% |
| 3 | 124 | 34.3% |
| Unknown | 6 | 1.7% |
| Stage |  |  |
| I | 147 | 40.6% |
| II | 170 | 47.0% |
| III | 45 | 12.4% |
| Hormone Receptor Status |  |  |
| ER+ and/or PR+ | 296 | 81.8% |
| ER/PR negative | 65 | 18.0% |
| Unknown | 1 | 0.3% |
| HER2 Status |  |  |
| Positive | 51 | 14.1% |
| Negative | 295 | 81.5% |
| Unknown | 16 | 4.4% |
| Regional Node Status |  | 0.0% |
| Positive | 149 | 41.2% |
| Negative | 213 | 58.8% |
| Type of Surgery |  |  |
| BCS | 189 | 52.2% |
| Mastectomy | 171 | 47.2% |
| No surgery | 2 | 0.6% |
| Recurrence |  |  |
| Local | 13 | 3.6% |
| Distant | 63 | 17.4% |
| No recurrence | 285 | 78.7% |
| Unknown | 1 | 0.3% |
| Vital Status |  |  |
| Death due to breast cancer | 50 | 13.8% |
| Death due to other cause | 42 | 11.6% |
| Alive | 268 | 74.0% |

Supplemental Table S2: Hazard ratios from multivariate analysis.

|  | **OS** | | **RFS** | |
| --- | --- | --- | --- | --- |
|  | **HR** | **P-Value** | **HR** | **P-Value** |
| **High centrosomes (vs low)** | 1.20 (0.76-1.90) | 0.427 | 1.25 (0.82-1.90) | 0.297 |
| **Stage II (vs I)** | 1.50 (0.87-2.60) | 0.143 | 1.69 (1.03-2.76) | 0.039 |
| **Stage III (vs I)** | 3.25 (1.69-6.24) | <0.001 | 3.30 (1.79-6.09) | <0.001 |
| **HR positive (vs negative)** | 0.43 (0.25-0.73) | 0.002 | 0.56 (0.34-0.92) | 0.021 |
| **HER2 positive (vs negative)** | 0.63 (0.33-1.18) | 0.151 | 0.68 (0.38-1.21) | 0.185 |
| **Grade 2 (vs 1)** | 1.38 (0.70-2.72) | 0.355 | 1.37 (0.75-2.53) | 0.308 |
| **Grade 3 (vs 1)** | 1.61 (0.77-3.37) | 0.207 | 1.68 (0.87-3.25) | 0.125 |

Supplemental Table S3: Sequences of primers used for qRT-PCR.

| Gene | Forward Sequence | Reverse Sequence |
| --- | --- | --- |
| *RRN18S* | GTAACCCGTTGAACCCCATT | CCATCCAATCGGTAGTAGCG |
| *GAPDH* | GGAGCGAGATCCCTCCAAAAT | GGCTGTTGTCATACTTCTCATGG |
| *ACTB* | CATGTACGTTGCTATCCAGGC | CTCCTTAATGTCACGCACGAT |
| *KRT7* | TCCGCGAGGTCACCATTAAC | GCTCTGTCAACTCCGTCTCAT |
| *KRT18* | GGCATCCAGAACGAGAAGGAG | ATTGTCCACAGTATTTGCGAAGA |
| *KRT19* | AACGGCGAGCTAGAGGTGA | GGATGGTCGTGTAGTAGTGGC |

**Supplemental Figure 1:** Distribution of average centrosome number per cell in the breast cancer patients represented in our TMA.


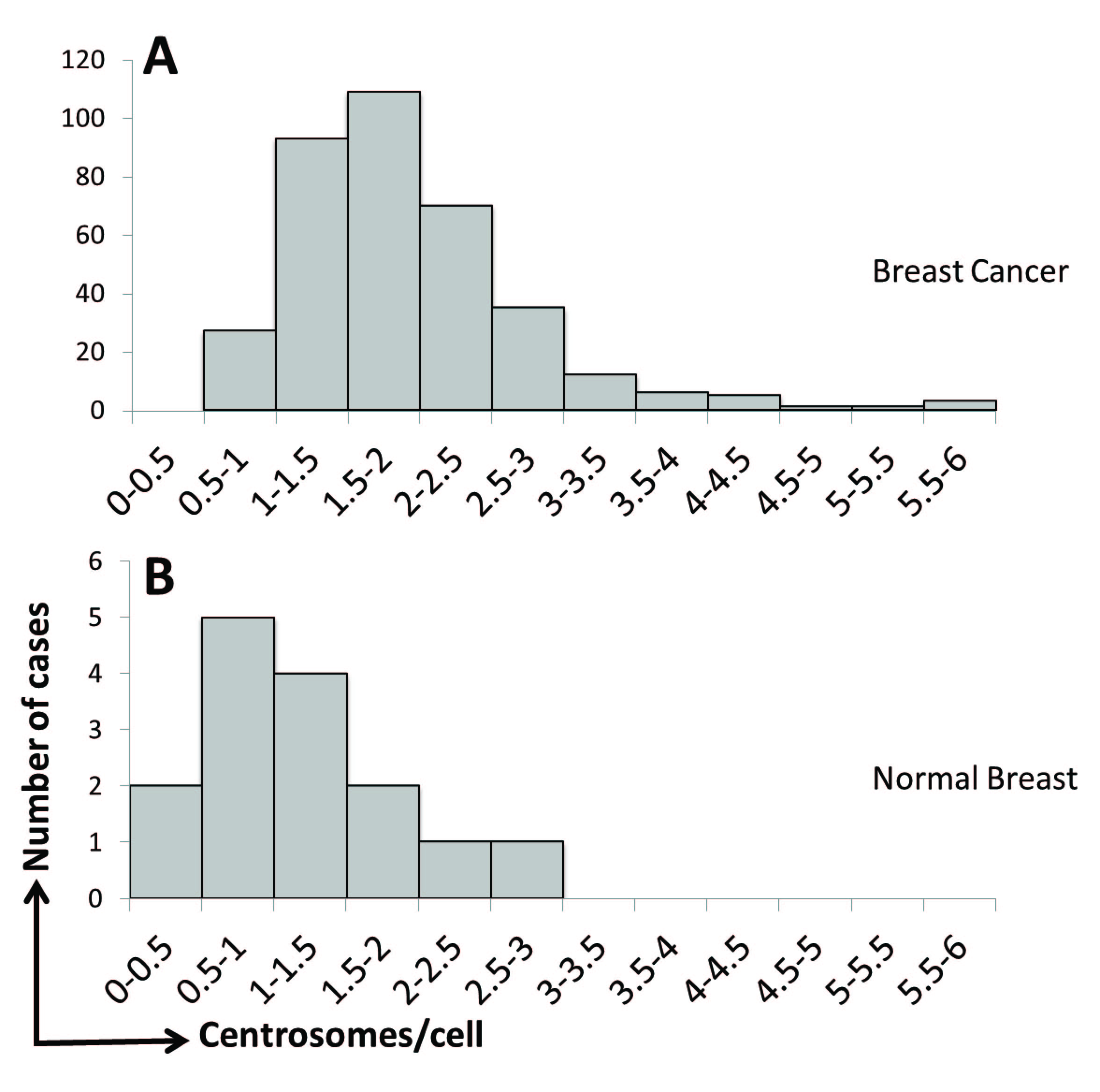


**Supplemental Figure 2: Correlations between centrosome amplification and nodal status, patient age, and tumor size.** CA was defined as an average of greater than 2 centrosomes (marked by pericentrin staining) per cell across the 3 tumor regions for each patient. (A) Bars represent percentage of patients with positive nodes ± standard error of proportion. (B-C) Bars represent average values ± SE.

**
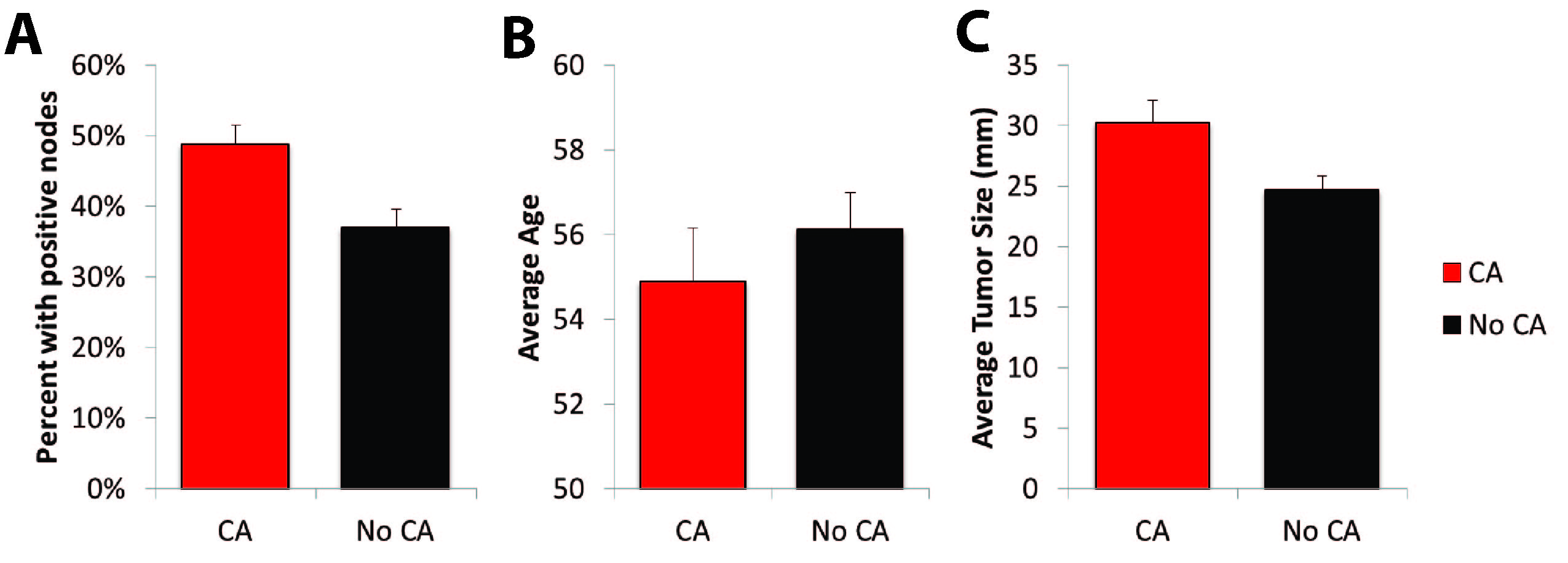
**

**Supplemental Figure 3: Centrosome clustering but not structural abnormalities correlate with worse outcomes in breast cancer.** Numbers and percentages in the left column indicate tumors containing centrosomes with atypical shapes, clustering, specking, and increased size. Tumors were considered to have atypical centrosome shapes if these were observed in more than 1 of the cells counted in at least 2 of the 3 tumor regions investigated; this same criterion was used for analysis of centrosome clustering and speckling. Clustering was defined as more than 2 distinct pericentrin foci together. Speckling was defined as more than 5 distinct pericentrin foci, which were smaller than typical centrosomes. For size, patients were divided into two groups (small versus large centrosomes) using the median centrosome size (0.99 μm) as the cutoff; therefore, half of cases fall into each group. Overall survival and recurrence-free survival are plotted using the Kaplan Meier method, and log rank tests were used to determine p values. Blue = DNA, green = pericentrin. Scale bar = 5 μm.

**
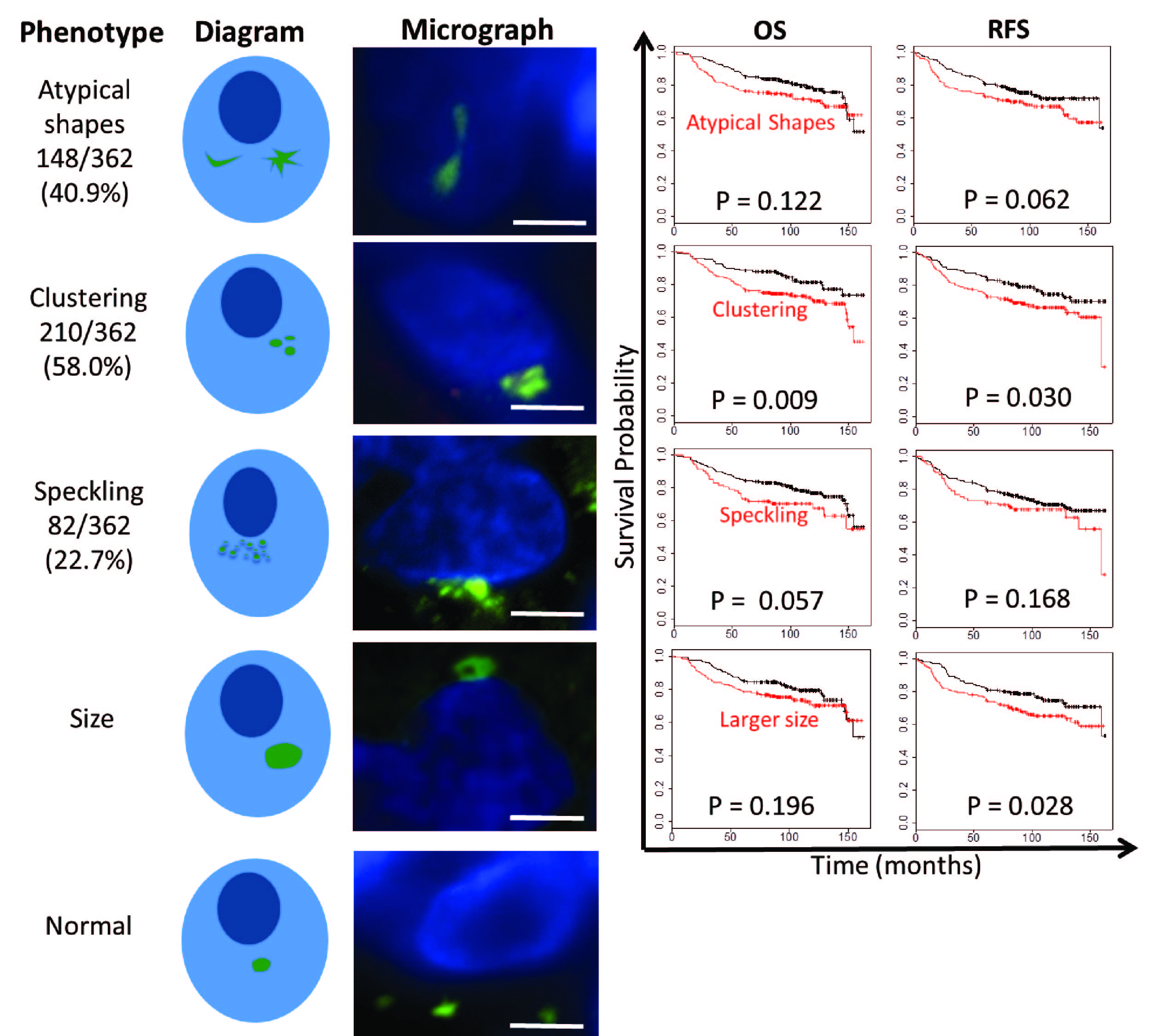
**

**Supplemental Figure 4: CIN is prognostic of worse breast cancer-related survival.** All-cause overall survival (A), recurrence-free survival (B), and breast cancer-specific survival (C) were assessed based on the presence of CIN. Displayed p-values are from log rank tests.

**
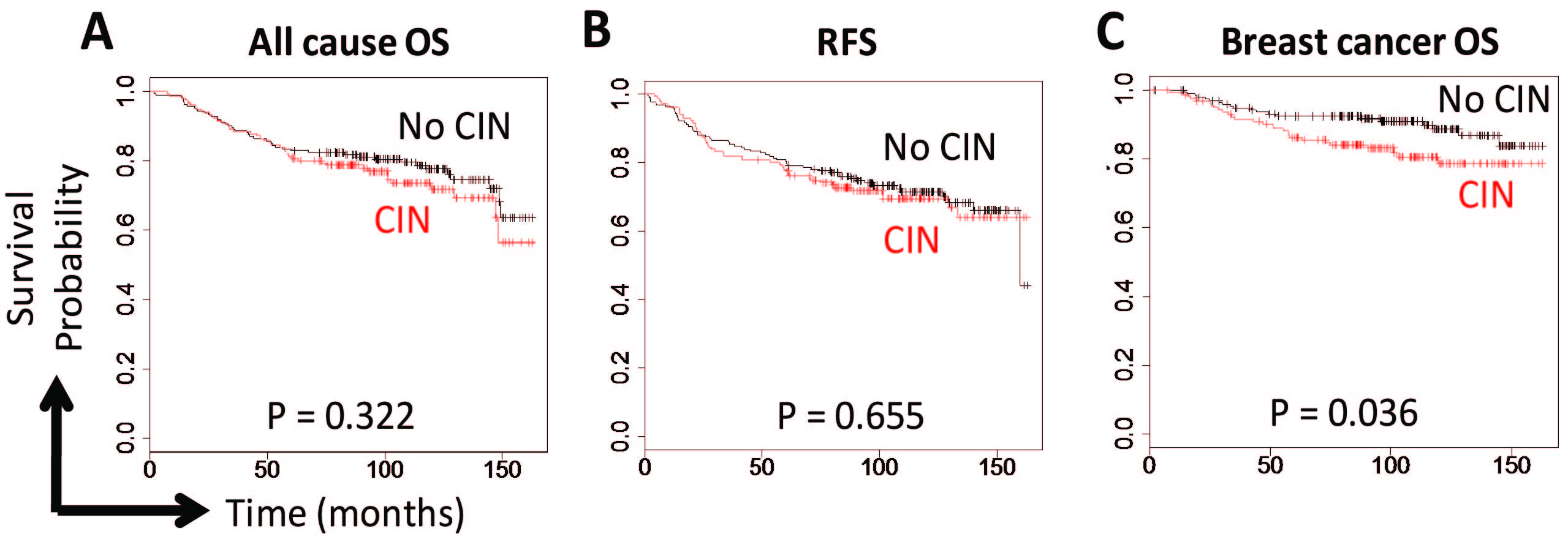
**

**Supplemental Figure 5: Centrosome amplification correlates with adverse clinical factors.** (A) These analyses used the overlap of pericentrin and polyglutamylated tubulin markers to define centrosomes. The pericentrin and polyglutamylated tubulin images are enlarged from the boxed region in the merged image. Scale bar = 2 μm. Blue = DNA, green = pericentrin, red = polyglutamylated tubulin. (B-D) Dot plots show the average centrosome number per cell in each tumor based on stage (B), grade (C), and subtype (D). Bars show averages ± SE. (E,F) Patients were divided into two groups (CA versus no CA) using an average of 2 centrosomes per cell as the cutoff. All-cause overall survival (E), recurrence-free survival (F), and breast cancer-specific survival (G) were assessed using the median centrosome value as a cutoff for low centrosomes versus high centrosomes. Displayed p-values are from log rank tests.

**
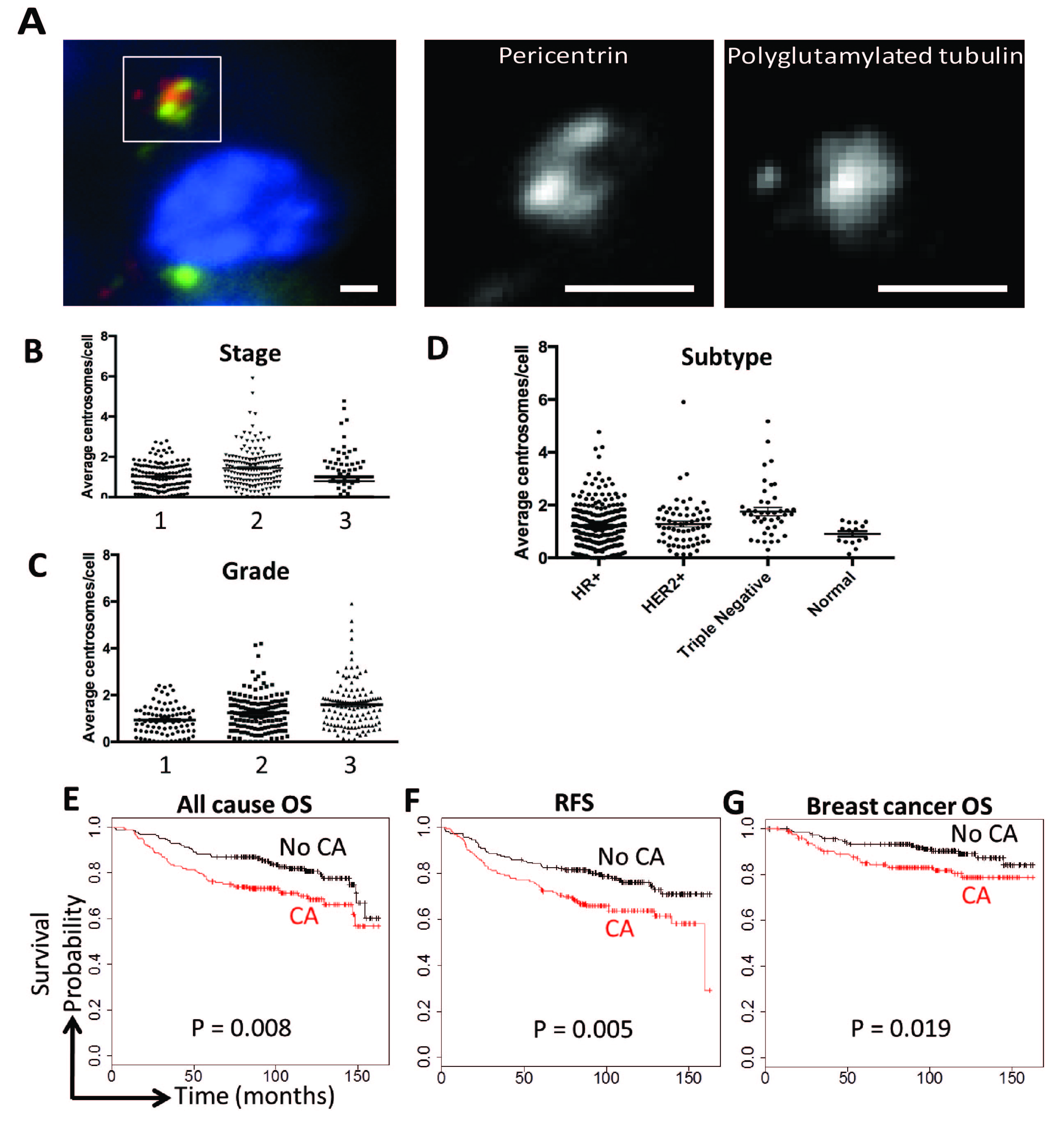
**

**Supplemental Figure 6: CA correlates with higher ploidy and CIN.** These analyses were performed by defining centrosomes as the overlap of pericentrin and polyglutamylated tubulin. (A, B) Patients were divided into two groups based on whether their average centrosome number was above or below 2. Ploidy was determined using 6-chromosome FISH. CIN was determined as the average non-modal chromosome number for each of the 6 chromosomes assessed by FISH. Bars indicate averages ± SE. (C,D) Scatterplots demonstrating the correlation of CA with ploidy and CIN.


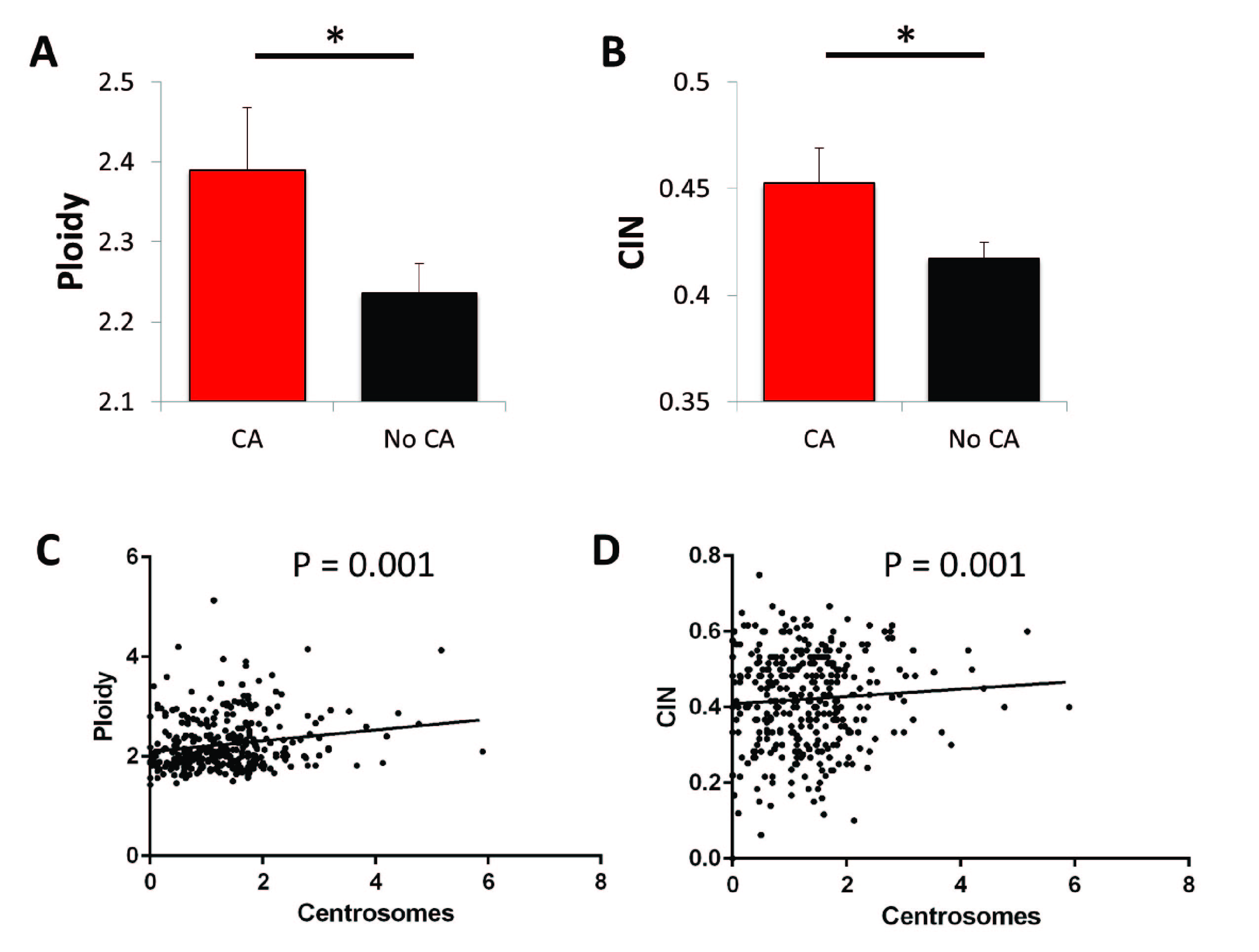

Supplement: Additional file 1: — Table S1. Patient characteristics. Table S2. Hazard ratios from multivariate analysis. Table S3. Sequences of primers used for qRT-PCR. Figure S1. Distribution of average centrosome number per cell in the breast cancer patients represented in our TMA. Figure S2. Correlations between centrosome amplification and nodal status, patient age, and tumor size. Figure S3. Centrosome clustering but not structural abnormalities correlate with worse outcomes in breast cancer. Figure S4. CIN is prognostic of worse breast cancer-related survival. Figure S5. Centrosome amplification correlates with adverse clinical factors. Figure S6. CA correlates with higher ploidy and CIN. (DOCX 6223 kb) [file 12885_2016_2083_MOESM1_ESM.docx]
